# Proteomic Characterization of Acyclovir-Induced Nephrotoxicity in a Mouse Model

**DOI:** 10.1371/journal.pone.0103185

**Published:** 2014-07-23

**Authors:** Hong Lu, Ya-Juan Han, Jia-Dong Xu, Wen-Min Xing, Jie Chen

**Affiliations:** School of Pharmacology, Zhejiang Chinese Medical University, Hangzhou, PR China; University of Texas Southwestern Medical Center, United States of America

## Abstract

Acyclovir (ACV) is an effective and widely used antiviral agent. However, its clinical application is limited by severe nephrotoxicity. We assessed ACV-induced nephrotoxicity and identified the differentially expressed proteins using mass spectrometry-based proteomic analysis. In total, 30 ICR mice were intraperitoneally administrated ACV (150 or 600 mg/kg per day) for 9 days. After administration of ACV, levels of serum creatinine and urea nitrogen increased significantly. In addition, mouse kidneys exhibited histopathological changes and reduced expression levels of vascular endothelial growth factor (VEGF) and its receptor VEGFR2. In the proteomic analysis, more than 1,000 proteins were separated by two-dimensional polyacrylamide gel electrophoresis, and a total of 20 proteins were up- or down-regulated in the ACV group compared with the saline group. Among these, six proteins (MHC class II antigen, glyoxalase 1, peroxiredoxin 1, αB-crystallin, fibroblast growth factor receptor 1-IIIb, and cytochrome c oxidase subunit Vb) were identified in association with ACV-induced nephrotoxicity. These findings were confirmed by Western blotting analysis. The differential expression levels of α-BC, Prx1, Glo I and CcO Vb suggest that oxidative damage and mitochondrial injury may be involved in ACV-induced nephrotoxicity. Furthermore, VEGF and FGF may play a role in tissue repair and the restoration process following ACV nephrotoxicity.

## Introduction

Acyclovir (or acyclovir) [9-(2-hydroxyethoxymethyl) guanine; ACV], is a synthetic purine nucleoside analog. It is widely used for the treatment of herpes simplex, herpes zoster, hepatitis B, and genital herpes. Recently, because of its extensive clinical application, reports of adverse drug reactions have increased. The main adverse reactions include nausea, vomiting, abdominal pain, diarrhea, dizziness, headache, urinary system damage, liver dysfunction, rashes, and allergic-like reactions. Therefore the Chinese State Food and Drug Administration has issued a drug use warning for ACV [1], [2].

ACV-induced nephrotoxicity mainly manifests as elevated plasma creatinine (Cr) and urea nitrogen levels, the occurrence of abnormal urine sediments, acute tubulointerstitial nephritis, and acute renal failure [3], [4]. The rate of ACV metabolism is very low *in vivo*, and the compound is mainly excreted by glomerular filtration and tubular secretion. Because of its low solubility in urine, rapid or excessive intravenous infusion can cause crystal precipitation and blockage in the kidney tubules, leading to acute renal failure [5], [6]. However, it has been reported that urea crystallization is a secondary factor in ACV renal toxicity – the main agent being the aldehyde metabolite of ACV, which can damage renal tubules directly [7].

Over the past decade, the use of proteomics in kidney disease research has grown substantially. Charlwood et al. [8] found significant changes in at least 20 proteins after injection of gentamicin, using proteomic techniques comprising two-dimensional polyacrylamide gel electrophoresis (2-DE), followed by *in situ* peptide N-glycosidase F (PNGase F) and trypsin digestion. The proteins identified were mainly involved in the citric acid cycle, gluconeogenesis, fatty acid biosynthesis, transportation, and the cellular stress response, indicating that impaired energy production and mitochondrial dysfunction may be associated with gentamicin renal toxicity. 2-DE and mass spectrometry (MS) were previously used by our team in an analysis of andrographolide sodium bisulfate (ASB)-induced nephrotoxicity, which revealed differential expression of six different proteins [9]. These findings suggested that oxidative stress in response to superoxide production in the mitochondria has an important role in renal toxicity induced by ASB treatment [9]. Therefore, proteomics technology is an important tool for studying the mechanism of drug-induced nephrotoxicity.

In the present study, changes in the protein expression profile accompanying renal impairment were evaluated in mice administered ACV for 9 days. In addition, we investigated the mechanism of ACV-induced nephrotoxicity and identified potential protein indicators using proteomics.

## Materials and Methods

### Animals and treatment

In total, 30 ICR mice were purchased from the Zhejiang Experimental Animal Center, China and randomly divided into three groups (n = 10 per group) to receive saline, 150 mg/kg ACV [2], [10], or 600 mg/kg ACV once-daily for 9 days. Mice were kept at room temperature (18–24°C) and 70±10% humidity, with a light-dark cycle (12 h–12 h). Food (Zhejiang Experimental Animal Center) and water were available *ad libitum*. ACV (Wuhan Humanwell Pharmaceutical Co, Ltd, Wuhan, China) was administered intraperitoneally (i.p.) diluted in 20 mL/kg of 0.9% sodium chloride injection solution (Huadong Pharmaceutical Co, Ltd, Hangzhou, China). The saline group received an equal volume of the 0.9% sodium chloride solution. All experimental procedures and protocols used in this study were reviewed and approved by the Zhejiang Chinese Medical University and were conducted in accordance with the Guide for the Care and Use of Laboratory Animals.

### Measurement of renal functions

On the 9^th^ day of treatment, mouse body weights were recorded and blood samples were obtained from the femoral arteries 1 h after the last injection, to measure serum levels of Cr and blood urea nitrogen (BUN). Blood samples were allowed to clot for 20 min at ambient temperature before centrifugation at 1,000 g for 10 min for serum separation. Levels of Cr and BUN were determined using commercially available kits (Nanjing Jiancheng Bioengineering Institute, Nanjing, China). The mice were then sacrificed by cervical dislocation and after exsanguination, the kidneys were extracted and weighed immediately. The renal index was calculated as kidney weight/body weight×100 (%). One kidney was used in the proteomic analysis; the other kidney was used for immunohistochemistry and histopathological assays.

### Histopathological examination

One kidney was fixed in neutral buffered formalin (pH 7.4) for more than 12 h, followed by paraffin embedding and sectioning at 3 um. The sections were then dewaxed with dimethylbenzene, stained with hematoxylin and eosin, and examined under a microscope (Olympus, Japan) at ×100 magnification.

### Quantitation of VEGF and VEGFR2 by immunohistochemistry

Kidney sections were washed three times (for 5 min each) with 0.01 mol/L phosphate-buffer saline (PBS; pH 7.4), followed by incubation for 10 min with 3% H_2_O_2_ in 0.01 mol/L PBS to prevent endogenous peroxidase activity. Then, sections were blocked for 1 h with 10% normal goat serum in 0.01 mol/L PBS and incubated overnight at 4°C with antibodies raised against VEGF (1∶200 dilution, Abcam, Massachusetts, USA) and VEGFR2 (1∶150 dilution, Abcam, Massachusetts, USA). Subsequently, the samples were incubated for 45 min at 37°C with goat anti-mouse or goat anti-rabbit IgG antibodies conjugated to HRP for detection. Finally, signals were visualized by the addition of 3,3′-diaminobenzidine tetrahydrochloride (DAB) and observed under a fluorescence microscope (Olympus). Signals were evaluated using a semi-quantitative scoring method: IRS  =  SI (positive intensity) × PP (the percentage of positive cells) [11]. SI levels were classified as follows: 0(cells were all negative), 1(weakly positive), 2(moderately positive), and 3(strongly positive). PP levels were classified as follows: 0(negative), 1(≤10%), 2(11∼50%), 3(51∼80%), and 4(>80%).

### Extraction of renal proteins

On the 9^th^ day of treatment, kidneys were surgically removed from mice in the saline and 600 mg/kg ACV groups for proteomic analysis. Protein extraction was performed as described previously [12]. Briefly, the kidney was dissected and the capsule removed. After washing three times with ice-cold saline (1∶10 w/v) to remove residual blood, the tissue was snap-frozen in liquid nitrogen, ground to a powder, and lysed in a buffer consisting of 30 mmol/L Tris, 8 mol/L urea, 4% (w/v) CHAPS and 10 mol/L PMSF (10 µL per 1 mL lysis buffer; Bio-Rad, USA), for 30 min at room temperature. Extracts were centrifuged for 5 min at 10,000×*g* at 4°C and the resulting supernatants were aliquoted and stored at −80°C. Finally, the protein concentrations were measured using the Micro BCA Protein Assay Kit (CWBIO, Beijing, China).

### 2-DE and MALDI-TOF-MS identification

Each sample containing 40 µg of protein was dissolved in 410 µL of rehydration solution (8.0 mol/L urea, 2% (w/v) CHAPS, 2.8% DTT, and 0.5% IPG buffer [pH 3–10], 0.002% (w/v) bromophenol blue), loaded onto 24 cm Immobiline Dry strips, pH 3–10 (GE Healthcare, USA). Isoelectric focusing (IEF) was performed on an Ettan IPGphor 3 IEF system (GE Healthcare) and focused for 12 h at 30 V, 1 h at 500 V, 1 h at 1,000 V, 1 h at 4,000 V, and finally for 8 h at 8,000 V. Following IEF, individual strips were equilibrated twice for 15 min in 50 mmol/L Tris-HCl, pH 8.8, 6 mol/L urea, 20% (v/v) glycerol, 2% (w/v) sodium dodecyl sulfate (SDS), 2% (w/v) DTT, and in a solution of the same composition that also contained 2.5% (w/v) iodoacetamide. The strips were immediately placed onto a 1 mm thick 12.5% (w/v) SDS polyacrylamide gel and sealed with 0.5% (w/v) agarose. SDS-PAGE electrophoretic separation was carried out for 45 min at 20 W and for 6 h at 80 W until the bromophenol blue dye reached the bottom of the gel.

After electrophoresis, the gel was stained with silver nitrate and spot scanning was performed using the UMAX Image Scanner and Lab Scan software. The stained protein spots of interest were excised from the gels and destained with 50 mmol/L Na_2_S_2_O_3_ and 15 mmol/L K_3_Fe (CN)_6_. Trypsin (5 µL at 12.5 ng/µL) was added to each sample for overnight digestion at 37°C. Then, the samples were analyzed on a matrix-assisted laser desorption/ionization tandem time-of-flight (MALDI-TOF) mass spectrometer (4800 Proteomics Analyzer; Applied Biosystems, USA). A combination of peptide mass fingerprints and peptide fragmentation patterns were used for protein identification in the NCBI non-redundant database using the Mascot search engine (www.matrixscience.com).

### Western blotting

Proteins (25 µg) were separated on 10% (w/v) SDS polyacrylamide gels (SDS-PAGE) and electrophoretically transferred onto PVDF membranes (Millipore, Bedford, MA, USA). Each membrane was blocked with 5% non-fat dried milk for 2 h and incubated overnight with primary antibodies against PRX1 (1∶200; Santa Cruz Biotechnology, USA), αB-crystalline (1∶400; Santa Cruz Biotechnology), and β-actin (1∶1,000; CWBIO, Beijing, China). After three washes with TBST, the blots were incubated with horseradish peroxidase-conjugated rabbit anti-goat and goat anti-mouse secondary antibodies at room temperature for 2 h. Signals were detected using the enhanced chemiluminescence kit (ECL; Cwbiotech, China). β-Actin was used as an internal loading control.

### Statistical analysis

Statistical analysis was performed using SPSS version 19.0 (SPSS Inc, Chicago, IL, USA). Data are reported as means±SD. Student's *t*-test or one-way ANOVA was used for statistical analysis of the differences in the original data between two groups, while Bonferroni correction was used in multiple testing. *P*-values less than 0.05 were considered statistically significant.

## Results

### Effect of ACV on body weight, renal index, and serum Cr and BUN levels

**Figure 1:**
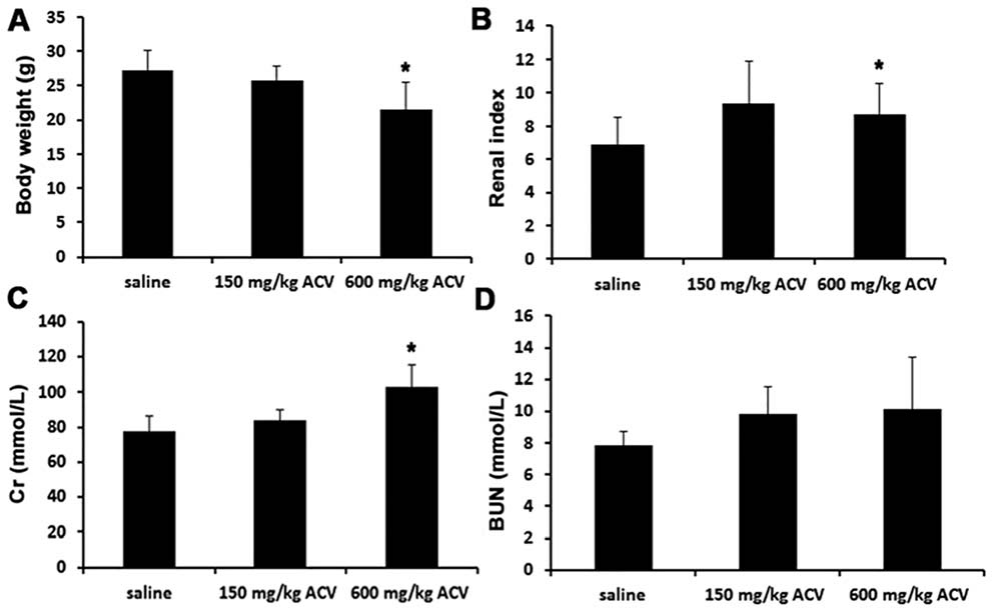
Effect of ACV on body weight, renal index, and serum levels of Cr and BUN. On the 9^th^ day of treatment, mouse body weights were recorded and blood samples were obtained from the femoral arteries 1 h after the last injection to measure the levels of serum creatinine (Cr) and blood urea nitrogen (BUN). (Means ± SD, n = 10), *P<0.05 vs. the saline group.

As shown in Figure 1, mouse body weights were reduced after administration of different amounts of ACV, whereas the renal index increased. In addition, the levels of Cr and BUN increased from 77.3±9.1 to 103±12.8 mmol/L and from 3.06±2.24 to 8.67±2.63 mmol/L, respectively, in mice administered 600 mg/kg ACV (*P*<0.05). Cr and BUN are important biochemical indicators of renal function and these findings confirmed ACV-induced nephrotoxicity.

### Renal histopathology

**Figure 2:**
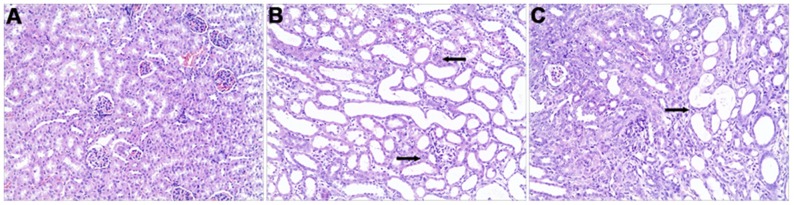
Effect of ACV on pathological changes in renal tissues of mice after administration of ACV for 9 days. Sections were stained with hematoxylin and eosin, and examined under a light microscope (HE×100, Olympus). (A) Saline group showing normal kidney morphology; (B) 150 mg/kg ACV group showing few inflammatory cells in the renal interstitium (arrows) and juxtaglomerular apparatus; (C) 600 mg/kg ACV group showing hyperplasia of fibrous tissue (arrow) and few inflammatory cells in the renal juxtaglomerular apparatus.

Mouse kidneys exhibited histopathological changes following ACV administration compared to the saline group. Figure 2 shows shedding and cloudy swelling of the brush borders of tubular epithelial cells, extensive tubular lumen expansion, tubular atrophy and hyperplasia, varying degrees of kidney interstitial infiltration by inflammatory cells, and fibrous tissue hyperplasia. No significant lesions were observed in the glomerular.

### VEGF and VEGFR2 expression

**Figure 3:**
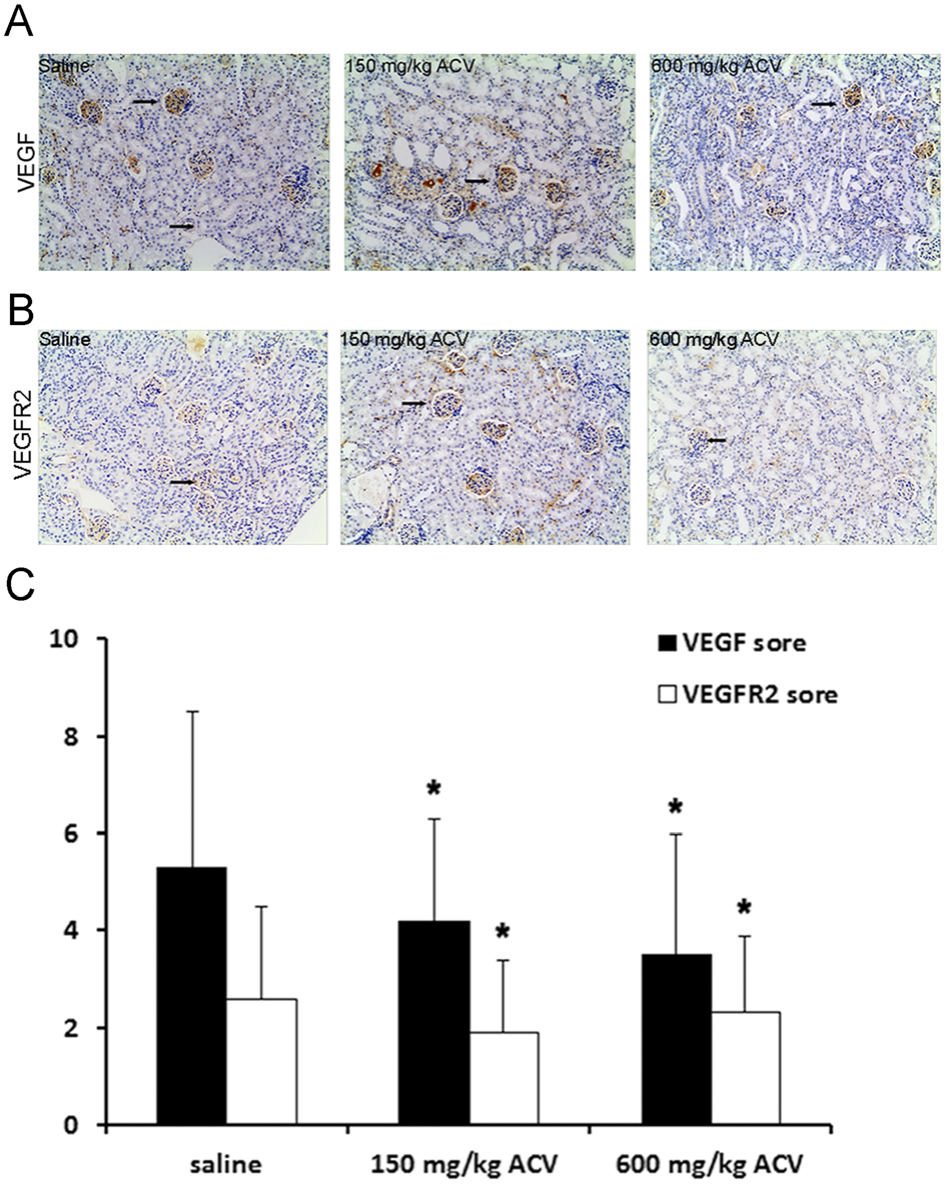
Effect of ACV on VEGF and VEGFR2 expression in mouse renal tissues. (A) VEGF expression in mouse kidney was assessed by immunohistochemical analysis after 9 days of saline or ACV administration (magnification ×100, arrows indicate glomerular and interstitial VEGFR2 expression). (B) VEGFR2 expression in mouse kidney was assessed by immunohistochemical analysis after 9 days of saline or ACV administration (magnification ×100, arrows indicate glomerular and interstitial VEGFR2 expression). (C) VEGF and VEGFR2 scores were calculated using the formula: IRS  =  SI (positive intensity) × PP (the percentage of positive cells) and were all significantly decreased after administration of ACV compared with saline group (means ± SD, n = 10), *P<0.05 vs. the saline group.

VEGF has an important role in renal fibrosis [13] and tissue repair [14]. According to clinical reports, ACV can induce acute kidney injury although the prognosis for recovery is good [1]–[4]. Due to the absence of reports describing ACV-induced fibrosis [1]–[4], we speculated that VEGF may participate in the kidney repair. VEGF and VEGFR2 expression was detected immunohistochemically and scores were calculated as described earlier. Data showed that the expression of VEGF and its receptor VEGFR2 in ACV groups was significantly reduced compared with the saline group *(P<*0.05; Fig. 3).

### 2-DE Gel separation and protein identification

**Figure 4:**
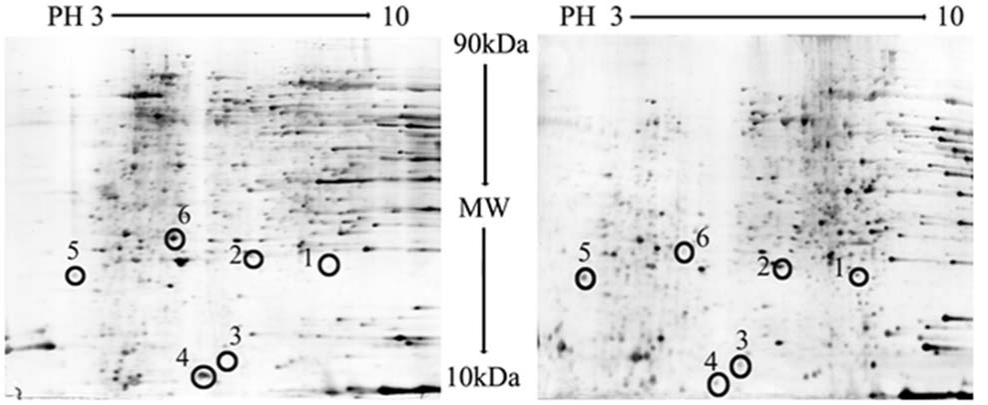
Two-dimensional electrophoresis gels of mouse renal tissues with (right) or without (left) 600 mg/kg ACV administered for 9 days. Proteins were separated in the first dimension by isoelectric focusing (pH 3–10) using Immobiline dry strips and in the second dimension using SDS-PAGE through 12.5% (w/v) acrylamide gels followed by silver staining. Differentially expressed proteins were excised from gels and identified using matrix-assisted laser desorption/ionization time-of-flight mass spectrometry.

The 2-DE analysis of the kidney proteome revealed up to 1,000 protein spots (Fig. 4). All spots were distributed in the region of PI 3–10 and had relative molecular weights of 10–100 kDa. Six differentially expressed protein spots were selected for MALDI-TOF analysis.

### Differential expression protein analysis

Six differentially expressed protein spots were identified after in-gel digestion, mass spectrometry and database searches (NCBInr). The characteristics of these proteins are listed in Table 1. Among the differentially expressed proteins, the levels of four (MHC class II antigen, glyoxalase I (Glo I), peroxiredoxin 1 (Prx1), and αB-crystallin (α-BC)) were increased, while the levels of two (fibroblast growth factor receptor 1-IIIb (FGFR1-IIIb), and cytochrome c oxidase subunit Vb (CcO Vb)) were significantly decreased.

**Table 1:** MALDI–TOF-MS identification of differentially expressed proteins identified in the NCBInr database.

Spot no	Protein	Accession No.	Protein MW (Da)	PI	Protein score	ACV effect	Fold change
1	Crystallin, alpha B	gi|6753530	20056.4	6.76	114	↑	14.5
2	Peroxiredoxin 1	gi|6754976	22162.3	8.26	108	↑	2.8
3	Glyoxalase I	gi|165932331	20796.4	5.24	57	↑	1.7
4	Cytochrome c oxidase, subunit Vb	gi|112807195	13837.9	8.34	39	↓	2.3
5	MHC class II antigen	gi|45751824	10940.3	5.42	45	↑	1.8
6	Fibroblast growth factor receptor 1-IIIb	gi|6175864	82070.3	6.29	48	↑	7.0

### Western blot analyses confirmation

**Figure 5:**
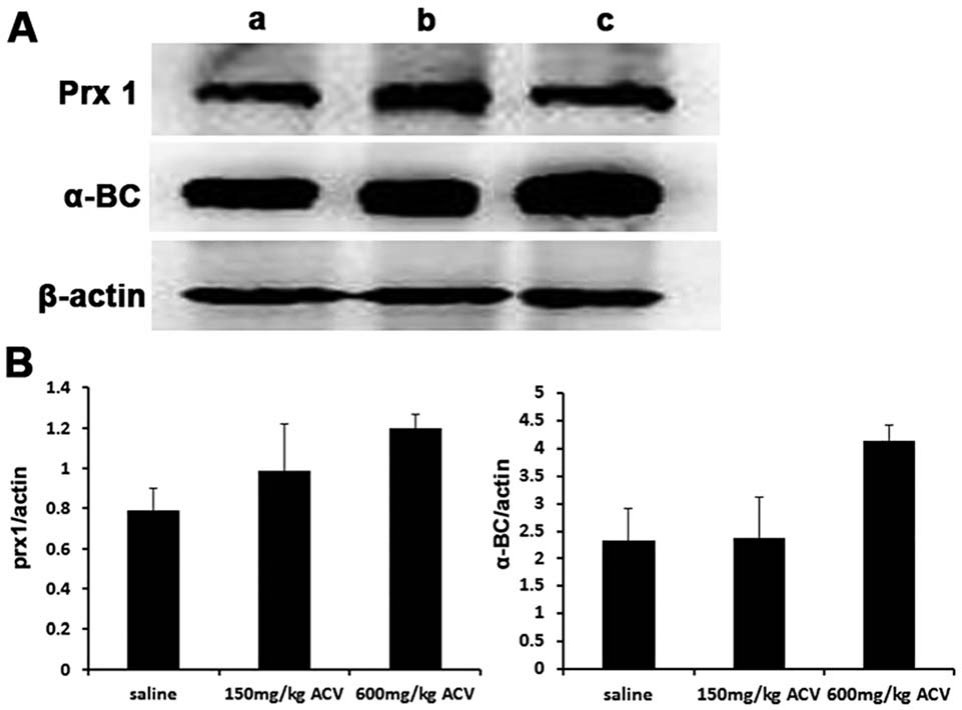
Western blot images and relative quantities of Prx1 and α-BC. (A) Prx1 and α-BC expression was detected by Western blotting in mouse kidneys after 9 days of saline or ACV administration (B) Relative quantities were defined as the ratio of the grayscale of Prx1 and α-BC to that of β-actin. *P<0.05 compared with the saline group.

Western blot analysis of two differentially expressed proteins (Prx1 and α-BC) was conducted to verify the proteomic analysis. The results indicated that the expression of Prx1 and α-BC in the ACV group was much higher than that in the saline group, which was consistent with the data obtained from the proteomic analysis (Fig. 5).

## Discussion

To examine whether ACV can induce nephrotoxicity, we administered a low dose of ACV (150 mg/kg, equivalent to the clinical dose used in humans) and a high dose (600 mg/kg, four times the clinical dose). As discussed, serum Cr and BUN levels increased significantly after 9 days of treatment with 600 mg/kg ACV, and mouse kidneys exhibited histopathological changes. Immunohistochemical analysis demonstrated markedly reduced expression of VEGF and VEGFR2. However, the effects of intermediate doses between 150 and 600 mg/kg require further study.

Because of the abundance of long-chain polyunsaturated fatty acids, the kidney is highly vulnerable to damage caused by reactive oxygen species (ROS) [15]. Oxidative stress is a deleterious condition that can cause cell damage and subsequent cell death due to oxidation of essential cellular components, such as DNA, lipids, and proteins [16]. The aldehyde metabolite of ACV causes direct damage to kidney tubules [7]. In animals, antioxidant and reactive oxygen scavengers are effective in protecting the kidney. Among the six differentially expressed proteins identified in the present study, three were oxidative stress response molecules; i.e., α-BC, Prx1, and Glo I, indicating that oxidative stress may have an important role in ACV-induced nephrotoxicity.

α-BC is the fifth member of the mammalian small heat-shock protein family (HspB5). Although isolated from the crystalline lens, it is widely distributed in brain, heart, skeletal muscle, and kidney. α-BC has chaperone-like functions and prevents the aggregation of denatured proteins after exposure to stresses such as heat-shock, oxidative stress, radiation, and anticancer drugs [17]. Importantly, α-BC is only upregulated when its cytoprotective properties are required [18]. As a cell stress protein, overexpressed α-BC can block the formation of ROS [19]. It has been reported that α-BC counteracts the mitochondrial apoptotic pathway, triggering the translocation of Bax and Bcl-Xs in the mitochondria, the release of mitochondrial cytochrome C in the cytosol, and the subsequent activation of downstream caspases including caspase-3 by inhibiting autoproteolytic maturation [20].

Peroxiredoxins (Prxs) are a novel family of small (22–27 kDa) nonseleno peroxidases. The major functions of Prxs include cellular protection against oxidative stress, regulation of cell proliferation, and modulation of intracellular signaling through H_2_O_2_ as a second messenger molecule. Six Prx isoforms (Prx1–6) have been identified [21], among which Prx1 is the most abundant and ubiquitously distributed in mammals [22]. This isoform is expressed at high levels in the kidney, liver, and small intestine [23]. The main function of Prx1 is to catalyze the reduction of H_2_O_2_ to maintain appropriate cellular redox levels. Along with its antioxidant activity, it also possesses anti-inflammatory and anti-atherogenic effects [24]. Cells deficient in Prx1 have increased sensitivity to oxidative DNA damage [25]. *Prx1* gene expression is elevated in numerous cancers [26], [27]. Prx1 upregulation in cells and tissues under oxidative stress represents a cellular recovery response to oxidative damage [28]. A study by Lee et al. showed that Prx has an important role in regulating 6-hydroxydopamine (6-OHDA)-induced apoptotic signaling. Overexpression of Prx1 prevented 6-OHDA-induced activation of p38 MAPK and subsequent activation of caspase-3 through scavenging of ROS, whereas knockdown of Prx1 enhanced most of these events [29]. In the present study, we observed an increase in Prx1 expression after ACV administration, suggesting that there is a correlation between oxidative stress and the nephrotoxicity of ACV. Prx1 is a key antioxidant enzyme in the kidney and could be important in detoxifying H_2_O_2_ generated during ACV toxicity.

The glyoxalase detoxification system has a major role in cellular defense against oxidative stress and glycation. Glo I, a cytosolic 42 kDa dimeric Zn^2+^ metalloenzyme, mainly participates in scavenging cytotoxic agents and metabolites (including free radicals) and detoxification [30]. Recent studies have shown that overexpression of Glo I can protect the kidney against ischemia and reperfusion injury by inhibiting the formation of intracellular methylglyoxal adducts and oxidative stress [31]. Therefore the upregulation of Glo I seen in the present study might represent an adaptive response to oxidative stress.

CcO Vb is essential for the assembly and stability of a functional cytochrome c oxidase complex. Hypoxia can cause a decrease in CcO Vb protein levels, which is accompanied by a decrease in enzyme activity [32]. Decreased cytochrome C oxidase activity has been shown to result from free radical injury or mitochondrial DNA damage [33], [34]. The expression of CcO Vb expression was markedly reduced in the present study, indicating that damage to mitochondrial respiration and dysfunction in oxidative phosphorylation may be involved in ACV-induced nephrotoxicity.

MHC class II has a central role in immune activation by presenting foreign peptides to antigen-specific T-helper cells and thereby inducing adaptive immune responses [35]. Enhanced MHC class II antigen expression in the kidney has been reported in both human and animal diseases [36], and has been well-documented in experimental ischemia/oxidative stress and kidney allograft rejection [37]. The significant increase in MHC class II antigen expression observed in this study suggests the upregulation of renal immunogenicity against ACV-induced nephrotoxicity.

The fibroblast growth factor (FGF) family consists of at least 22 peptide growth factors [38] that are involved in many biological processes, including pattern formation, cellular differentiation, mitogenesis, angiogenesis, and wound-healing [39]. FGFR1-IIIb, a 100–130 kDa glycosylated protein, is found at moderate levels at the cell membrane and cytoplasm, and expressed preferentially in the dermis and the epidermis of mouse tail skin and brain. The binding of FGF to FGFR leads to dimerization and tyrosine autophosphorylation of the docking protein FGF receptor substrates (FRS) 2a and -b, resulting in recruitment of multiple Grb2–Sos complexes and activation of the MAPK signaling pathway [40]. Similarly, VEGF can bind to VEGFR2 and activate Ras, thereby further activating Raf-1 and stimulating MAPK signaling cascades, resulting in caspase-3 and caspase-7-mediated apoptosis via the intrinsic and extrinsic pathways, respectively [41]. VEGF and FGF are both closely associated with angiogenesis and tissue repair [42], [43]. According to clinical reports, ACV induces acute kidney injury but with a good prognosis and there are no reports of ACV-induced fibrosis [1]–[4]. We speculate that down-regulation of VEGF and FGF in the ACV groups may have an important role in kidney repair. How the regulation of FGF and VEGF expression in association with ACV-induced renal injury and restoration requires further study.

## Conclusion

We investigated the protein profile of ACV-induced toxicity using proteomics. The differential expression levels of α-BC, Prx1, Glo I and CcO Vb suggest that oxidative damage and mitochondrial injury may be involved in ACV-induced nephrotoxicity. Furthermore, VEGF and FGF may play a role in tissue repair and the restoration process following ACV nephrotoxicity.
